# Openness, Integrity, Inclusion, and Innovation in Scholarly Communication: Competing or Complementary Forces?

**DOI:** 10.3389/frma.2021.767869

**Published:** 2022-01-03

**Authors:** Virginia Barbour

**Affiliations:** Office for Scholarly Communication, Queensland University of Technology (QUT), Brisbane, QLD, Australia

**Keywords:** open access, integrity, infrastructure, COVID-19, preprint, Open Science, innovation

## Introduction

In 2020, the importance of open and rapid communication of academic research came to the fore, as possibly never before, in the global effort to address the COVID-19 pandemic. The pandemic arrived at a time when much of the infrastructure for sharing research openly and rapidly was already in place, and to a large extent, the global publishing enterprise was able to fulfill its function of dissemination of information.

However, we are already seeing signs that publishing may revert to a more closed model post pandemic. It is also clear that the pandemic has exacerbated some of the problems in scholarly communication, such as a worsening participation by women and unequal distribution of funding globally. Furthermore, it is not clear that some of the innovations developed in the pandemic for sharing of information—such as the CORD-19 dataset of publications—will endure in their current state. Finally, the sheer volume of publishing, especially through relatively novel mechanisms, such as preprints, has led to uncertainty about how to support trust in research publications, both in the academic community and in the wider public.

## Infrastructure and Ideas Ready for a Pandemic

The COVID-19 pandemic that emerged in 2020 and which at the time of writing is still ongoing led to probably the biggest disruption in scholarly communication seen since academic publishing began to move online at the end of the 20th century. What has been critical to the success thus far of much of this disruption is that it builds on emerging infrastructure and ideas that have primed the publishing system for change.

There are previous examples of publishing having to respond to a global medical emergency; the most recent relevant of these is SARS in 2003 (SARS | Basics Factsheet | CDC, n.d). That emergency was fortunately relatively short lived, and although the global medical research community rose to the challenge of investigating SARS, the global publishing community barely coped. A 2010 analysis showed that of the research done during the SARS global emergency, the majority of it was published well after the emergency was over: only 22% of the studies were submitted, 8% accepted, and 7% published during the epidemic of Xing et al. (2010). The contrast with the COVID-19 pandemic could not be clearer. There has been an outpouring of research, and most of this research is rapidly and freely available online, in the first instance predominantly on preprint servers (Fraser et al., 2021). Although preprint servers have a long history in some disciplines, until the COVID-19 pandemic their use and indeed their very acceptability in medical publishing were untested. *medRxiv*, founded by the BMJ and Yale University (Rawlinson and Bloom, 2019) in association with Cold Spring Harbor Press, the founders of *bioRxiv*, has perhaps been the standout success for publishing in the pandemic. Launched just before the pandemic, it was perfectly placed to support the publishing effort required but saw its submissions rise from a few hundred in 2019 (Bloom, 2020) to more than 13,000 preprints related to the pandemic to date (medRxiv, n.d) At the height of the pandemic, it was seeing millions of views per month of its content.

By the standards of preprints, more traditional publishing lagged far behind. Further, until a concerted call from a global coalition of government scientists and policy advisors, led by the Office of Science and Technology at the White House, it was not even clear under what terms research would be made available in the pandemic (Call to Action to the Tech Community on New Machine Readable COVID-19 Dataset – The White House, n.d.). The results of that call, the CORD-19 database, is another example of infrastructure in waiting. The key to the success of CORD-19 was its alignment with a set of key principles—the FAIR principles, originally described for data, which have come to have huge importance in the research data world. FAIR principles, which require high-quality metadata such as permanent identifiers and licenses, facilitate discoverability, machine readability, and interoperability, allowing sophisticated text mining and reuse of the research literature (Wang et al., 2020).

## Will Open Access to Research Endure Post Pandemic?

At the time of writing in July 2021, the pandemic continues globally with no obvious end date in sight. The rate of publication has decreased from the peaks of 2020, but the need for research to remain open remains in the face of coronavirus mutations and continuing societal challenges. Despite this, we are beginning to see publishers moving papers behind subscription barriers in a move that is reminiscent of publisher activities in earlier medical emergencies, such as following Ebola outbreaks. These moves illustrate clearly the stranglehold that traditional publishers retain over the dissemination of research publications. It reinforces the need for research publications to be fully open at the time of publication and that is only done by ensuring that articles are openly licensed with Creative Commons licenses. More generally, the pandemic has reinforced the need for a diversity of approaches to publishing models (Shearer et al., 2020) as well as a robust open infrastructure as championed by organization such as Invest in Open (Invest in Open Infrastructure, n.d) to support these models.

## Who Lost Out in Pandemic Publishing?

The pandemic also laid bare many of the entrenched inequalities in scholarly communication, and indeed in research more generally. Money was poured into research globally on every possible aspect of the pandemic, from basic science such as genomic sequencing, through to analyses of public health. However, as in research in more normal times, the money did not flow equitably, nor were publications from the pandemic truly reflective either of research needs or of the wider researcher landscape. For women, the COVID-19 pandemic “exacerbated pre-existing gender inequity in the STEM workforce across the Asia-Pacific region” according to a 2021 report (Impact of COVID-19 on Women in the STEM Workforce | Asia-Pacific, n.d.) which further noted that “Additional domestic responsibilities, such as supervising school learning at home, caused competing priorities as domestic roles and professional roles overlapped. This resulted in negative impacts on productivity for many women, especially in terms of academic output such as journal publications.” Nor were research projects on COVID-19 equally distributed globally. A summary of COVID-19 Funding Trends 2021 noted that “90 per cent of research projects are located in high income countries, with the greatest number in the US.” (Special Report, 2021)

## Trust, Integrity, and Rewards in Research

The pandemic also exacerbated many of the trends in relation to trust—or lack of trust—in research. The rapid availability by necessity of non-peer-reviewed research in the form of preprints and the intense public interest and wide sharing of research through the news media triggered an intense discussion on trust in research. Confidence in research was highlighted by analysis that showed that the pandemic led to “a proliferation of research projects underpowered and unable to achieve their aims” (Norton et al., 2021) but which nonetheless were eagerly pored over and discussed widely. As traditional publishers tried to keep up with the flood of papers, it was notable that some of the most egregious examples of poor-quality research were actually published in high-profile peer-reviewed journals, which had apparently failed in proper scrutiny of research, especially in relation to access to underlying data (Two Elite Medical Journals Retract Coronavirus Papers over Data Integrity Questions | Science | AAAS, n.d.).

In some ways then, the pandemic also accelerated conversations about how to assess research for trustworthiness and how to balance speed of sharing versus scrutiny through peer review—which as is well known is, at best, an imperfect and partial way to assess the quality of research publications. *medRxiv*, which had to develop processes on the fly for the rapid screening of preprints, has, as a result, of the pandemic now a quality control process that, although no substitute for peer review, does seem able to reliably filter out research which has ethical or similar issues. The increase in the amount of research available as preprints—and the scrutiny of this publishing approach—has also led to a wider understanding in the press and wider public arena of what peer review means, and it is common to see now that news reports will indicate the peer review status of reported research. Furthermore, publishing of research through preprints challenges one of key norms of research evaluation, which is currently overwhelmingly biased to rewarding researchers for publishing in specific journals. The new models of publishing can only accelerate discussions on the urgent need for reform of the incentive system as championed by DORA (Declaration on Research Assessment, n.d) and others.

## Building a Better Future for Research Sharing

So what comes after the pandemic? In many ways, the pandemic has acted as an accelerator for discussions about open access and open science that previously had been caught up in bureaucratic niceties. In May, this year UNESCO provisionally agreed the text of its Open Science Recommendation (UNESCO Recommendation on Open Science, 2020). Its origin in 2019 was pre-pandemic, but by the time of its release the topic could not be more timely and its preamble referenced the pandemic as follows: “Noting that the global COVID-19 health crisis has proven worldwide the urgency of fostering an equitable access to scientific information, facilitating the sharing of scientific knowledge, data and information, enhancing scientific collaboration and science- and knowledge-based decision making to respond to global emergencies and increase the resilience of societies.”

Further international work on open science inspired by the pandemic included an online UN Open Science meeting in July 2021 (United Nations. Open Science Conference, 2021) with more than 2,500 participants with global perspectives on the role of open science in the pandemic, and what lessons need to be learned from the pandemic in addressing the overarching emergency of our time, climate change. The clear consensus was that we cannot reverse the open research and sharing practices that have come to be normalized during the pandemic if we are going to collaborate effectively to combat climate change. In his keynote speech, Prof. Geoffrey Boulton highlighted the report of the International Science Council “Opening the record of science: making scholarly publishing work for science in the digital era” which calls for an urgent and robust reform of the scholarly publishing process according to the following seven principles:

1) There should be universal open access to the record of science, both for authors and readers.
2) Scientific publications should carry open licenses that allow reuse and text and data mining.
3) Rigorous and ongoing peer review is essential to the integrity of the record of science.
4) The data/observations underlying a published truth claim should be concurrently published.
5) The record of science should be maintained to ensure open access by future generations.
6) Publication traditions of different disciplines should be respected.
7) Systems should adapt to new opportunities rather than embedding inflexible infrastructures.

This last principle is perhaps the most important—the need for constant adaptation as needed. If there is one critical lesson that we have learned over the 18 months of the pandemic and which will surely be further reinforced before the pandemic is over, it is that previous models of publishing and research dissemination—in particular our reliance on proprietary publishing and infrastructure and associated incentive structures based solely on publication in specific journals—can no longer be considered fit for purpose.

## References

[B1] Bloom T. (2020). Shepherding Preprints Through a Pandemic. BMJ. 371, m4703. 10.1136/bmj.m4703 33323377

[B2] Call to Action to the Tech Community on New Machine Readable COVID-19 Dataset – The White House (n.d). Retrieved August 2, 2021, Available at: https://trumpwhitehouse.archives.gov/briefings-statements/call-action-tech-community-new-machine-readable-covid-19-dataset/ .

[B3] Declaration on Research Assessment (n.d). DORA. Retrieved August 31, 2021, Available at: https://sfdora.org/ .

[B4] Fraser N. Brierley L. Dey G. Polka J. K. Pálfy M. Nanni F. (2021). The Evolving Role of Preprints in the Dissemination of COVID-19 Research and Their Impact on the Science Communication Landscape. Plos Biol. 19 (4), e3000959. 10.1371/journal.pbio.3000959 33798194 PMC8046348

[B5] Impact of COVID-19 on women in the STEM workforce | Asia-Pacific. (n.d). 89.

[B6] Invest in Open Infrastructure (n.d). Invest in Open Infrastructure. Retrieved August 31, 2021, Available at: https://investinopen.org/ .

[B7] medRxiv (n.d). medRxiv.org—The Preprint Server for Health Sciences. Available at: https://www.medrxiv.org/ (Accessed July 31, 2021).

[B17] Norton A. Bucher A. Antonio E. Mounier P. (2021). A living mapping review for COVID-19 funded research projects: nine-month update [version 4; peer review: 2 approved]. Wellcome Open Res. 5, 209. 33117894 10.12688/wellcomeopenres.16259.1 PMC7579366

[B8] Rawlinson C. Bloom T. (2019). New Preprint Server for Medical Research. BMJ. 365, l2301. 10.1136/bmj.l2301 31167753

[B9] SARS | Basics Factsheet | CDC (n.d). Retrieved August 2, 2021, Available at: https://www.cdc.gov/sars/about/fs-sars.html .

[B16] Shearer K. Chan L. Kuchma I. Mounier P. (2020). Fostering Bibliodiversity in Scholarly Communications: A Call for Action. Zenodo. 10.5281/zenodo.3752923

[B10] Special Report: Covid-19 Funding Trends (2021). Research Professional News. Available at: https://www.researchprofessionalnews.com/rr-news-world-special-report-covid-19-funding-trends/ .

[B11] Two elite medical journals retract coronavirus papers over data integrity questions | Science | AAAS (n.d). Retrieved August 2, 2021, Available at: https://www.sciencemag.org/news/2020/06/two-elite-medical-journals-retract-coronavirus-papers-over-data-integrity-questions .

[B12] UNESCO Recommendation on Open Science (2020). The UNESCO Open Science Recommendation was adopted on 23rd November 2021. Available at: https://en.unesco.org/science-sustainable-future/open-science/recommendation .

[B13] United Nations. Open Science Conference (2021). United Nations; United Nations. Available at: https://www.un.org/en/library/OS21 (Accessed August 31, 2021).

[B14] Wang L. L. Lo K. Chandrasekhar Y. Reas R. Yang J. Burdick D. (2020). CORD-19: The COVID-19 Open Research Dataset. ArXiv:2004.10706 [Cs]. Available at: http://arxiv.org/abs/2004.10706 .

[B15] Xing W. Hejblum G. Leung G. M. Valleron A.-J. (2010). Anatomy of the Epidemiological Literature on the 2003 SARS Outbreaks in Hong Kong and Toronto: A Time-Stratified Review. Plos Med. 7 (5), e1000272. 10.1371/journal.pmed.1000272 20454570 PMC2864302

